# Genome‐wide mapping and prediction of plant architecture in a sorghum nested association mapping population

**DOI:** 10.1002/tpg2.20038

**Published:** 2020-08-07

**Authors:** Marcus O. Olatoye, Zhenbin Hu, Geoffrey P. Morris

**Affiliations:** ^1^ Department of Agronomy Kansas State University Manhattan KS 66506 USA; ^2^ Current address: Department of Crop Science University of Illinois at Urbana‐Champaign Urbana IL 61801 USA

## Abstract

Modifying plant architecture is often necessary for yield improvement and climate adaptation, but we lack understanding of the genotype‐phenotype map for plant morphology in sorghum. Here, we use a nested association mapping (NAM) population that captures global allelic diversity of sorghum to characterize the genetics of leaf erectness, leaf width (at two stages), and stem diameter. Recombinant inbred lines (*n* = 2200) were phenotyped in multiple environments (35,200 observations) and joint linkage mapping was performed with ∼93,000 markers. Fifty‐four QTL of small to large effect were identified for trait BLUPs (9–16 per trait) each explaining 0.4–4% of variation across the NAM population. While some of these QTL colocalize with sorghum homologs of grass genes (e.g., those involved in transcriptional regulation of hormone synthesis [rice *SPINDLY*] and transcriptional regulation of development [rice *Ideal plant architecture1*]), most QTL did not colocalize with an a priori candidate gene (92%). Genomic prediction accuracy was generally high in five‐fold cross‐validation (0.65–0.83), and varied from low to high in leave‐one‐family‐out cross‐validation (0.04–0.61). The findings provide a foundation to identify the molecular basis of architecture variation in sorghum and establish genomic‐enabled breeding for improved plant architecture.

**Abbreviations**
AES: Additive effect size
BLUP: Best linear unbiased predictor
FE: Family effect
FLT: Flowering time
HGT: Plant height
LFE: Leaf erectness
NAM: Nested association mapping
NFE: No family effect
PLW: Preflag leaf width
PVE: Proportion of variation explained
QTL: Quantitative trait loci
RIL: Recombinant inbred line
SNP: Single nucleotide polymorphism
STD: Stem diameter
VLW: Vegetative leaf width.

## 1 INTRODUCTION

Modification of plant architecture during crop improvement has greatly contributed to global agricultural productivity during the last century (Duvick, 2005; Khush, 2001). Ideotype breeding involves the creation of a model plant with characteristics that facilitate efficient photosynthesis, growth, and yield (Donald, 1968; Messina, Hammer, Dong, Podlich, & Cooper, 2009). The Green Revolution ideotypes targeted in maize, rice, and wheat include reduced height, erect leaves, thick stalks, large and semi‐compact inflorescence (ear, panicle, or head) (Khush, 2001). Several plant architecture traits have been under investigation for ideotype breeding. Leaf erectness (i.e., the angle between the culm and leaf midrib) is thought to have contributed to increased grain yield in maize through adaptation of hybrids to high planting densities (Duvick, 2005; Hammer et al., 2009). Wide leaves may facilitate efficient solar radiation capture for increased plant productivity (Sarlikioti, de Visser, Buck‐Sorlin, & Marcelis, 2011). Thick stems may increase stalk strength and improve standability (Kashiwagi, Togawa, Hirotsu, & Ishimaru, 2008).

Genetic variation for these architectural traits exists in cereals that can be utilized in crop improvement (Jia et al., 2013; Tian et al., 2011; Zhao et al., 2011). Variation in aboveground plant structures may be due to genetic differences in regulators of maintenance, determinacy, and structure in apical meristem (Pautler, Tanaka, Hirano, & Jackson, 2013). The leaves and shoots are generated from the shoot apical meristem at the vegetative phase, while the inflorescence meristems differentiate after floral transition (Wang & Li, 2008). Some key regulatory genes underlying plant architecture in grasses are *liguleless* genes (*lg1*–*lg4*) (Johnston et al., 2014; Moreno, Harper, Krueger, Dellaporta, & Freeling, 1997), *brachytic2* (*br2*) (Multani et al., 2003), *drooping leaf* (*drl1/drl2*) (Strable et al., 2017), and *Ideal Plant Architecture1 (IPA1)* (Liu et al., 2019; Miura et al., 2010). Growth hormones such as auxins, gibberellins, and brassinosteroids also regulate plant architecture (Wang & Li, 2008). Notably, major plant architecture regulators often have pleiotropic effects on other traits (such as inflorescence shape, yield components, and disease resistance) that can enhance or diminish their utility for crop improvement (Ishii et al., 2013; Kim et al., 2018; Liu et al., 2019).

Understanding trait genetic architecture is essential to design effective breeding strategies (Bernardo, 2008). Genetic architecture can be characterized by various quantitative trait loci (QTL) mapping approaches, each with strengths and weaknesses (Morrell, Buckler, & Ross‐Ibarra, 2012). While most crop trait dissection has used biparental populations, the effectiveness of these studies were generally limited by small population size and low genetic diversity. Association mapping exploits wide genetic diversity, but is limited by population structure that causes spurious and synthetic associations (Korte & Farlow, 2013). Nested association mapping (NAM) reduces the confounding effect of historical population structure and phenotypic variation through crosses among founder lines (Yu, Holland, McMullen, & Buckler, 2008). Thus, NAM is an efficient approach to characterize the genetic basis of complex traits, especially adaptive traits that may be confounded with population structure (Bouchet et al., 2017). In addition, the NAM approach has been used to explore genomic selection in maize (Peiffer et al., 2014) and barley (Maurer et al., 2015) and investigated as a potential training population for breeding (Rincent, Charcosset, & Moreau, 2017).

Sorghum is an important staple food crop in semi‐arid and arid regions globally due to its resilience to challenging environmental conditions (Monk, Franks, & Dahlberg, 2014). Sorghum cultivation in temperate climates during the last century was made possible through conversion of tall photoperiod‐sensitive tropical germplasm to semi‐dwarf photoperiod‐insensitive lines (Klein et al., 2008). Genetics of flowering time and plant height in global sorghum diversity has been well characterized (Bouchet et al., 2017; Burks, Kaiser, Hawkins, & Brown, 2015; Mace, Hunt, & Jordan, 2013; Morris et al., 2013; Thurber, Ma, Higgins, & Brown, 2013; Upadhyaya, Wang, Gowda, & Sharma, 2013). However, the genetics of vegetative architecture traits such as leaf morphology and stem diameter are much less understood (Feltus et al., 2006; Mantilla Perez, Zhao, Yin, Hu, & Fernandez, 2014; Shehzad & Okuno, 2015; Zhao, Mantilla Perez, Hu, & Salas Fernandez, 2016). Natural variation for plant architecture has been well characterized in maize, a relative of sorghum in tribe Andropogoneae, and found to be controlled by loci of small effect (a predominantly polygenic architecture) (Li et al., 2015; Tian et al., 2011). However, evolutionary genetic theory suggests that sorghum, as a predominantly selfing species (Barnaud, Trigueros, McKey, & Joly, 2008), would be expected to have a greater proportion of moderate to large effect variants than maize, an outcrossing species (Griswold, 2006).

**Core Ideas**

- Understanding the genetics of plant architecture could facilitate the development of crop ideotypes for yield and adaptation.
- The genetics of plant architecture traits was characterized in sorghum using multi‐environment phenotyping in a global nested association mapping population.
- Fifty‐four quantitative trait loci were identified; some colocalize with homologs of known developmental regulators, but most do not.
- Genomic prediction accuracy was consistently high in five‐fold cross‐validation, but accuracy varied considerably in leave‐one‐family‐out predictions.

In this study, we characterized the genetic basis (location of QTL, number of QTL, effect sizes) of vegetative morphology traits in sorghum using a large NAM population and tested the accuracy of genomic prediction. Our results suggest that natural variation for vegetative traits in sorghum is under the control of a few loci of moderate effect and many loci of small effect (i.e., variation has oligogenic and polygenic components). While some QTL colocalized with homologs of maize and rice candidate genes, most did not, suggesting that natural variation for vegetative morphology in sorghum may be due predominantly to genes not previously described in other cereals.

## 2 MATERIALS AND METHODS

### 2.1 Plant materials and phenotypic evaluation

The sorghum NAM population consists of 2200 recombinant inbred lines (RILs) derived from a cross between a common founder line RTx430 (an important U.S. pollinator parent line) and 10 other diverse founder lines chosen to capture a wide genetic and morphological diversity (Supplemental Table S1) (Bouchet et al., 2017). The genotyping of the population and the genome‐wide SNP data set were previously described (Bouchet et al., 2017; Hu, Olatoye, Marla, & Morris, 2019). The population was phenotyped in multiple environments in Kansas, US (Table 1) for leaf erectness, leaf width, stem diameter, flowering time and height. Leaf erectness (LFE) was defined as the angle between the pre‐flag leaf midrib and a line perpendicular to culm at the point of pre‐flag leaf attachment, and measured using a barcode reader and barcoded protractor (Supplemental Figure S1). Leaf erectness was measured from three plants per plot. The leaf width was measured as the width of the leaf at the widest point on both pre‐flag leaf and the fourth leaf from the flag leaf on three plants per plot (Supplemental Figure S1). Measurements were taken using a barcode‐scanning ruler. Stem diameter was measured as the diameter of the stem at the second internode above the ground, on three plants per plot, using a digital caliper. Flowering time was scored as the number of days from planting to the day in which 50% of the individuals in a plot are in anthesis. Inflorescence architecture phenotypes were previously described (Olatoye et al., 2020).

**TABLE 1:** Multi‐environmental phenotyping of sorghum nested association mapping population

Location	Climate	Year	Annual Precipitation (mm)^a^	Environment Code	Traits^b^
Manhattan, KS	Humid Continental	2014	698	MN14	LET, PLW, VLW, STM, FLT
Hays, KS	Semi‐Arid	2014	639	HI14	STM, HGT
Hays, KS	Semi‐Arid	2015	513	HI15	LET, VLW, PLW, HGT

^a^Precipitation from October of the preceding year to October of the given year.

^b^Phenotypic traits evaluated are Leaf erectness (LET), pre‐flag leaf width (PLW), vegetative leaf width (VLW), stem diameter (STM), height (HGT), and flowering time (FLT).

### 2.2 Phenotypic data analysis

Analysis of phenotypic data collected was performed using LME4 R package (Bates, Mächler, Bolker, & Walker, 2015). The phenotypic mean of each RIL across environments was estimated. Genomic heritability for each trait was calculated by dividing genetic variance component by the sum of genetic variance and residual variance component obtained using *mixed.solve* function in the rrBLUP R package (Endelman, 2011). Pairwise Pearson correlation among traits were estimated using the residuals derived from fitting a linear model for family and trait phenotypic means:

(1) $$y_{ijk}=\mu +\gamma_{i}+\varepsilon_{ij}$$

where *y_ij_* is the phenotype; $\gamma_{i}$ is the term for the NAM families; and $\varepsilon_{ij}$ is the residual term, for the *j‐*th individual in the *i‐*th family. The BLUPs (best linear unbiased predictors) for each phenotype was estimated by fitting the term for RILs (genotypes), environment, and RILs by environment interactions as random effects.

### 2.3 Joint linkage mapping

Joint linkage mapping was performed using single environment phenotypes and trait BLUPs using the stepwise regression approach implemented in TASSEL 5.0 (Glaubitz et al., 2014). The approach is based on forward inclusion and backward elimination stepwise methods. The entry and exit limit of the forward and backward stepwise regressions were set at 0.001. The threshold cutoff was set at 1.8 × 10^−6^ based on 100 permutations. The genotypic data used for this analysis have been previously described (Hu et al., 2019). The JL model was specified as:

(2) $$y=\;b_{0}+\;\alpha_{f}u_{f}+\;\sum_{i=1}^{k}x_{i}b_{i}+\;e_{i}$$

where *b*_0_ is the intercept; *u_f_* is the effect of the family of the founder *f* obtained in the cross with the common parent (RTx430); α*_f_* is the coefficient matrix relating *u_f_* to **y**; *b_i_* is the effect of the *i‐*th identified locus in the model; *x_i_* is the incidence vector that relates *b_i_* to **y**; and *k* is the number of significant QTL in the final model.

Estimation of QTL effect size and allele frequency using the *calc_snp_stat* function from https://github.com/ekfchan/evachan.org-Rscripts. While the proportion of phenotypic variation explained (PVE) by each marker was estimated by fitting a regression model with terms for family and QTL nested within family as fixed effects (Würschum et al., 2012):

(3) $$y_{ijk}=\mu +\gamma_{i}+\omega_{ij}+\varepsilon_{ijk}$$

where *y_ijk_* is the phenotype; $\gamma_{i}$ is the family term; $\omega_{ij}$ is the term for QTL nested within family *i*; and $\varepsilon_{ijk}$ is the residual. The sum of squares of marker nested within family divided by the sum of squares total gave the proportion of variance explained by the detected QTL. The additive effect size of the QTL in the population was estimated as the average of the difference between the mean phenotypic values associated with the two‐allele class of the QTL. The additive effect size of each QTL was estimated relative to the common parent RTx430.

### 2.4 Comparison of QTL regions with a priori candidate genes

A list of a priori candidate genes was developed for leaf morphology and plant architecture based on homology with genes underlying these traits in other cereals (Supplemental File 1). The list included 25 maize genes, 20 rice genes, and two sorghum genes (47 sorghum genes in total) known to control plant architecture. The corresponding putative sorghum orthologs were derived from the Phytozome “gene ancestry” feature, which is inferred based on sequence similarity and synteny (Goodstein et al., 2012). A custom R script was used to search for a priori genes that colocalized with the QTL within a window of 250 kb, the average distance until linkage disequilibrium decays to *r^2^* < 0.1 in the NAM population (Hu et al., 2019). An enrichment analysis of a priori genes around identified QTL was carried out using a chi‐squared test. The frequency of colocalization of QTL with a priori genes was compared to the null expectation, that is, colocalization of QTL with random genes from the sorghum genome (from the Phytozome version 3.1 gff3 file).

### 2.5 Genomic predictions

Genomic predictions were performed using the *mixed.solve* function in the ridge regression best linear unbiased prediction (rrBLUP) package in R (Endelman, 2011). The prediction models is as follows:

$$y=\mu +\;\sum_{m=1}^{p}Z_{m}u_{m}+e$$

where **y** is the vector (*n* × 1) of observations; μ is the vector of the general mean; *p* is the number of markers (*p* > *n*); **Z** *_m_* is the *m‐*th column of the design matrix **Z;** *u_m_* is the genetic effect associated with *m*‐th marker; and *e* is the residual. Genomic prediction was cross‐validated using two approaches. First, the “leave‐one‐family‐out” prediction approach was performed by removing each family's genotypic and phenotypic data and using the remaining nine families to predict the given family. A five‐fold cross validation approach was used where the entire NAM population was divided into *n* = 5 equal groups, then *n* − 1 groups at a time were used to train the model to predict the remaining one. The process was then repeated for 100 cycles. For the five‐fold cross validation, two types of response variables were used in the genomic prediction model. First, the phenotypic mean across environments (no‐family‐effect), and second the residuals from the regression of trait values and family effect (family‐effect).

## 3 RESULTS

### 3.1 Phenotypic variation for plant architecture traits

**FIGURE 1:**
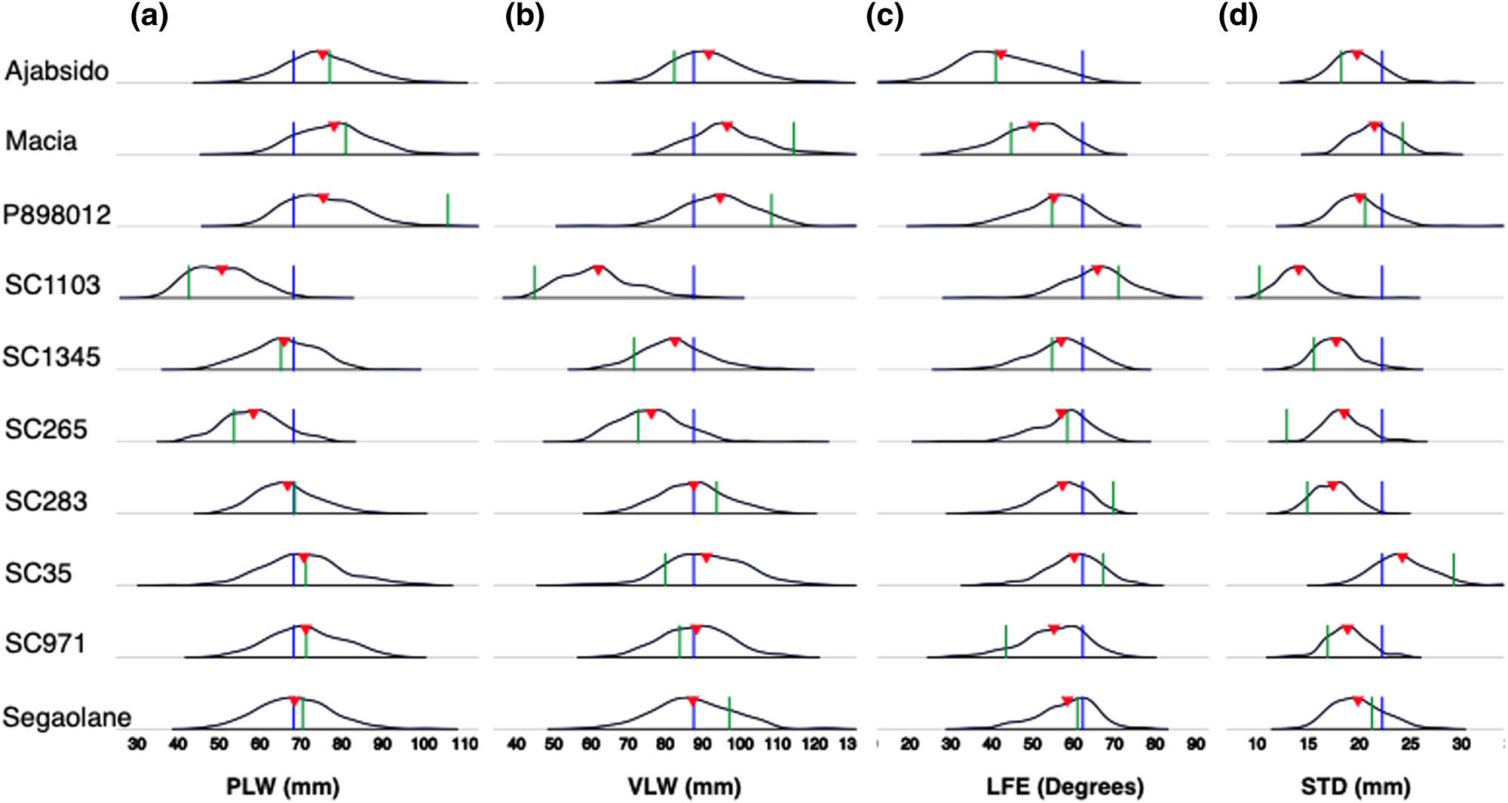
Phenotypic distribution of traits within sorghum nested association mapping (NAM) families. Density plots showing phenotypic distributions for (a) preflag leaf width (PLW), (b) vegetative leaf width (VLW; fourth leaf below the flag leaf) (c) leaf erectness (LFE) of the preflag leaf, and (d) stem diameter (STD). Triangles indicate the mean trait value in each family, green lines indicate the trait value of the diverse founder for each family, and blue lines indicate the trait value of the common founder

Phenotypic observations were made on 2200 RILs in ten NAM families in three environments (year‐by‐location), resulting in 35,200 data points for vegetative traits. Phenotypic distribution of vegetative traits varied substantially among families (Figure 1; Table 2). Mean PLW ranged from 51 mm in the SC1103 family to 78 mm in the Macia family; mean VLW ranged from 62 mm in the SC1103 family to 97 mm in the Macia family; mean LFE ranged from 42 deg in the Ajabsido family to 66 deg in the SC1103 family; and mean STD ranged from 14 mm in the SC1103 family to 24 mm in the SC35 family. Phenotypic distributions were predominantly symmetrical, both with given families and with respect to the parent phenotype (Figure 1). In a few cases, RIL families had asymmetrical distributions (with respect to parent phenotypes) for some traits (e.g., LFE in the Ajabsido family, PLW in the SC1103 family).

**TABLE 2:** Summary of phenotypic and genotypic variation

Trait^a^	Range	Mean	*h* ^2^ ^b^	Range of PVE (%) for QTL^c^	Range of AES for QTL^d^
HGT (cm)	53–270	109	0.53	0.2–14.7	−12–7
FLT (d)	45–101	64	0.56	0.3–9.3	−4–4
STM (mm)	10–34	19	0.43	0.4–1.6	−2–3
LFE (deg)	13–85	56	0.36	0.6–4.1	−3–7
PLW (mm)	33–112	68	0.31	0.4–2.6	−6–6
VLW (mm)	45–131	86	0.25	0.5–1.2	−5–8

^a^Preflag leaf width (PLW), vegetative leaf width VLW, leaf erectness (LFE), stem diameter (STD), plant height (HGT), and days to flowering time (FLT).

^b^Genomic heritability (*h* **^2^**).

^c^Phenotypic variation explained.

^d^Additive effect size.

**FIGURE 2:**
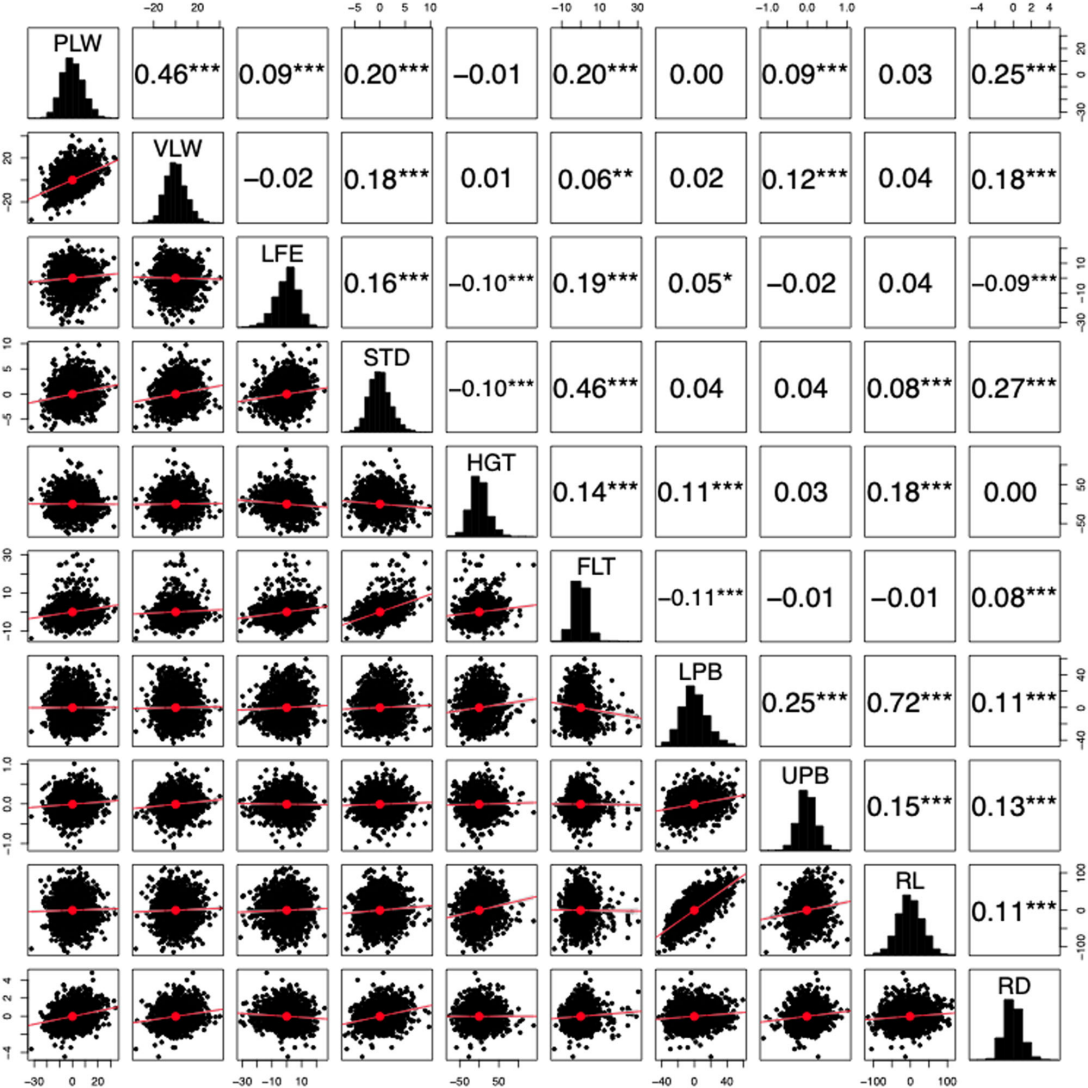
Relationships among plant architecture traits. Pairwise Pearson correlation among traits (preflag leaf width [PLW], mm), vegetative leaf width (VLW, mm), leaf erectness (LFE, degrees), stem diameter (STD, mm), plant height (HGT, cm), flowering time (FLT, d), lower primary branch (LPB, mm), upper primary branch (UPB, mm), rachis length (RL, mm), and rachis diameter (RD, mm) after accounting for family effect (correlation of residuals of a linear model with a fixed family term). The correlation values shown are significant at *** *P*‐value < .001, ** *P*‐value < .01, and * *P*‐value < .05

The relationship among pairs of traits, estimated as pairwise phenotypic correlations, differed substantially among trait pairs (Figure 2). LET had a weak negative correlation (*r* = −0.10; *P*‐value < .01) with height and positive correlation with FLT (*r* = 0.19; *P*‐value < .01) and STD (*r* = 0.16; *P*‐value < .01), respectively. PLW was positively correlated with VLW (*r* = 0.46; *P*‐value < .01), as expected, and had the same positive relationship with both STD and FLT (*r* = 0.20; *P*‐value < .01). STD was positively correlated with FLT (*r* = 0.46; *P*‐value < .01) and slightly negatively correlated to HGT (*r* = −0.09; *P*‐value < .01). Environmental correlation among each trait ranged from 0.50 (*P*‐value < .01) for VLW (MN14 and HI15) to 0.66 (*P*‐value < .01) for STD (MN14 and HI14) (Supplemental Figure S2).

### 3.2 Identified QTL and their effect sizes

**FIGURE 3:**
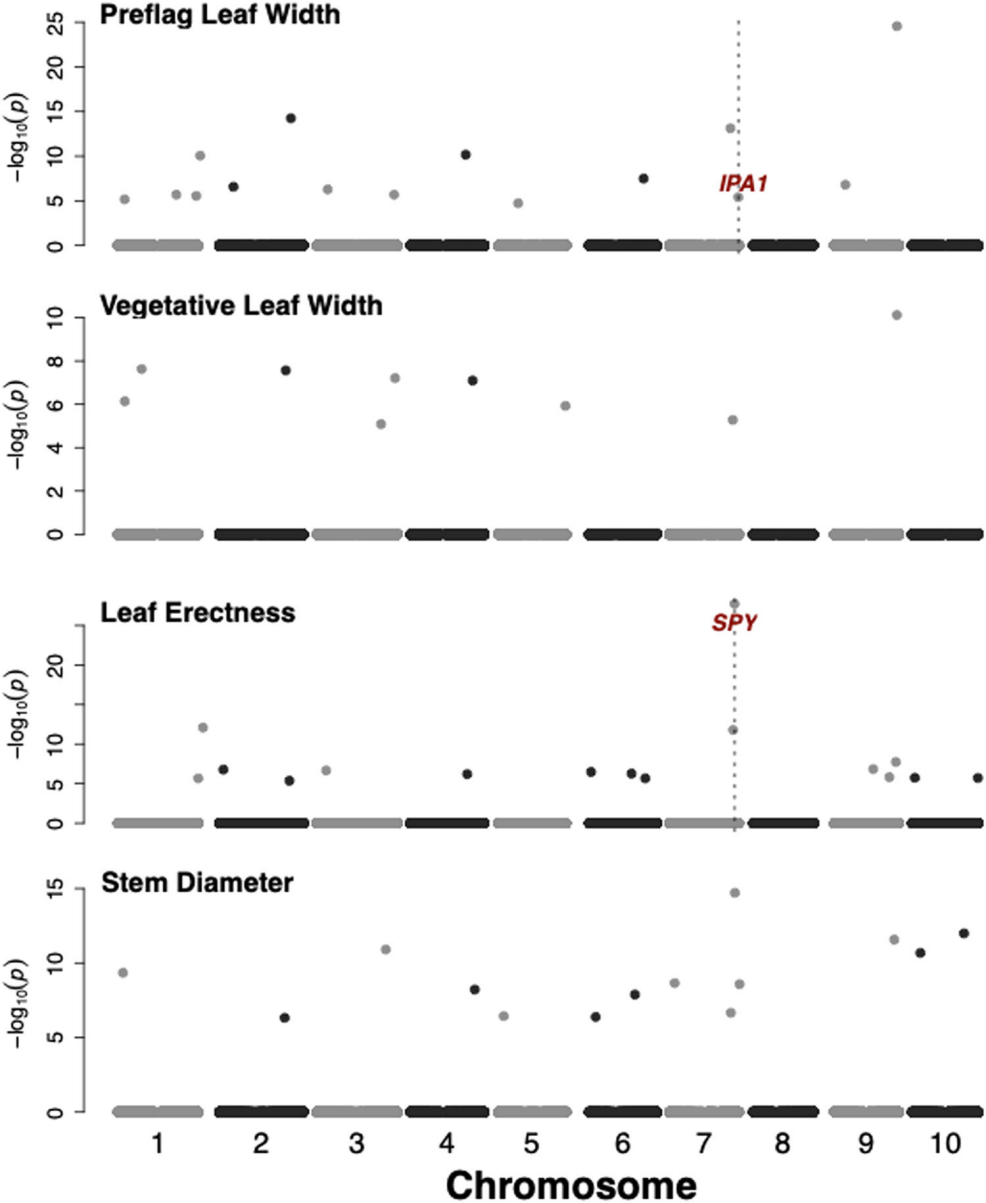
QTL mapping for vegetative morphology trait BLUPs using joint linkage model. Genomic location of associations with best linear unbiased predictors of (a) preflag leaf width, (b) vegetative leaf width, (c) leaf erectness, and (d) stem diameter. A priori candidate genes that colocalize within 250 kb of QTL are noted, with putative orthologs of genes of interest in brown

Joint linkage analysis was performed to characterize the genetic architecture of the four vegetative morphology traits (Tables 2 and 3; Figure 3). Significant associations were observed for all traits, both in single environments and for trait BLUP across environments, with 60 QTL across all traits in single environment analysis (Supplemental File 2), and 54 QTL across all traits for BLUPs (Table 3). For trait BLUP associations, 16 SNPs explained 19% of LFE variation, 15 SNPs explained 15% of PLW variation, 9 SNPs explained 9% of VLW variation, and 14 SNPs explained 13% of STD variation. Allele frequencies of the QTL range from 0.05 to 0.45 for LFE, 0.05 to 0.39 for PLW, 0.10 to 0.33 for VLW, and 0.05 to 0.48 for STD. In general, the associated loci were of small to moderate effect when considering the proportion of variance explained across the entire NAM population. Among all traits, SNP S7_60258739, associated with LFE, explained the largest proportion of variation (4%). Among the 54 trait‐associated SNPs, 50% of them (27 SNPs) explained less than 1% of phenotypic variation for the given trait.

**TABLE 3:** Markers associated with trait BLUPs in the NAM population

Trait^a^	Marker^b^	QTL	PVE^c^	AES^d^	MAF^e^	Gene ID	Sorghum Homolog	Distance (kb)
LFE	S7_60258739	qLFE7.6026	4.1	6.5	0.12	*SPY*	Sobic.007G168300	60
	S7_58924868	qLFE7.5892	2.6	5.3	0.11			
	S1_78036446	qLFE7.0804	1.6	−0.7	0.31			
	S10_60475045	qLFE10.6048	1.1	0.7	0.45			
	S6_38758657	qLFE6.3876	1.1	−2.7	0.22			
	S1_73639818	qLFE1.7364	1	0.6	0.2			
	S2_3899853	qLFE2.0390	1	−0.8	0.45			
	S3_8738913	qLFE3.0874	1	−1.1	0.23			
	S2_64697782	qLFE2.6470	0.9	0.7	0.23			
	S6_1570949	qLFE6.0157	0.9	−5	0.09			
	S10_2514655	qLFE10.0251	0.7	−0.5	0.05			
	S4_52117037	qLFE4.5212	0.7	−0.5	0.14			
	S6_51419656	qLFE6.5142	0.7	−1.4	0.1			
	S9_35638689	qLFE9.3564	0.7	2.7	0.12			
	S9_56496135	qLFE9.5650	0.7	3.3	0.25			
	S9_50511159	qLFE9.5051	0.6	−0.8	0.05			
PLW	S9_57434878	qPLW9.5743	2.6	−5.5	0.32			
	S1_75424764	qPLW1.7543	2	−3.5	0.31			
	S2_66035565	qPLW2.6604	1.9	−1.6	0.39			
	S1_71800701	qPLW1.7180	1	−2.9	0.1			
	S3_71232350	qPLW3.7123	1	1.4	0.38			
	S7_56472059	qPLW7.5647	1	−4	0.13			
	S3_10099179	qPLW3.1010	0.8	−0.2	0.14			
	S4_50769836	qPLW4.5077	0.8	1	0.19			
	S6_49869080	qPLW6.4987	0.8	1.4	0.14			
	S2_13170804	qPLW2.1317	0.7	−4.1	0.22			
	S7_63675831	qPLW7.6368	0.7	2.9	0.3	*IPA1*	Sobic.007G210200	202
	S5_18331282	qPLW5.1833	0.6	3.3	0.16			
	S9_10093897	qPLW9.1009	0.6	−0.1	0.05			
	S1_53491169	qPLW1.5349	0.5	1	0.05			
	S1_5993169	qPLW1.0599	0.4	6	0.06			
STD	S6_41904494	qSTD6.4190	1.6	−0.2	0.33			
	S7_60620622	qSTD7.6062	1.3	0.4	0.17			
	S7_64856654	qSTD7.6486	1.2	−0.2	0.33			
	S2_60290585	qSTD2.6029	1.1	0.02	0.39			
	S3_63584041	qSTD3.6358	1.1	−1.5	0.13			
	S1_4379437	qSTD1.4379	1	1.1	0.34			
	S6_5734088	qSTD6.0573	1	−0.9	0.07			
	S4_59019567	qSTD4.5902	0.9	−1.1	0.32			
	S9_54968356	qSTD9.5497	0.9	0.04	0.48			
	S10_7615967	qSTD10.0762	0.8	1	0.27			
	S10_47679206	qSTD10.4768	0.7	−1.4	0.06			
	S7_5137535	qSTD7.0514	0.7	−1	0.24			
	S5_5038559	qSTD5.0504	0.4	−0.7	0.05			
	S7_56802286	qSTD7.5680	0.4	3	0.05			
VLW	S3_59217270	qVLW3.5922	1.2	−0.1	0.27			
	S3_72108998	qVLW3.7211	1.2	0.1	0.33			
	S9_57434698	qVLW9.5744	1.2	−5.2	0.32			
	S2_61314023	qVLW2.6131	1.1	−2.5	0.28			
	S7_58607155	qVLW7.5861	1.1	−3.7	0.25			
	S4_57114581	qVLW4.5712	0.9	−5.1	0.27			
	S5_61851015	qVLW5.6185	0.9	7.7	0.1			
	S1_21577664	qVLW1.2158	0.8	3.2	0.26			
	S1_6040251	qVLW1.0604	0.5	2.8	0.11			

^a^Preflag Leaf Width (PLW), Vegetative Leaf Width (VLW), Leaf Erectness (LFE), and Stem Diameter (STD).

^b^Marker name provides the chromosome number before the underscore and position after the underscore

^c^PVE: Proportion of variation explained.

^d^AES: Additive Effect Size estimated in the direction of RTx430 allele in original trait units.

^e^MAF: Minor allele frequency.

^f^Gene ID: Abbreviated gene name for the a priori candidate gene that colocalizes with the QTL (within 250 kb).

### 3.3 QTL colocalized with plant and inflorescence morphology genes

Some a priori candidate genes colocalized with a QTL for single‐environment or BLUP traits (5 of 47; Figure 3; Table 3; Supplemental Table S1). For the single‐environment analysis of STD, a significant SNP (S7_59787744; PVE = 2%; MAF = 0.18) colocalized with an a priori candidate gene: the sorghum auxin efflux transporter gene (Sobic.007G163800; *Dwarf3*) at a distance of 34 kb. In addition, a significant SNP for STD (S10_8142199; PVE = 1.2%; MAF = 0.22) was 42 kb from the sorghum ortholog (Sobic.010G091700) of the rice *Brassinosteroid Upregulated1* (*BUL1*) gene. For LET, a significant SNP (S7_63865966; PVE = 2%; MAF = 0.19) was 12 kb from the putative sorghum ortholog (Sobic.007G210200) of rice *Ideal Plant Architecture1* gene (*IPA1*). Also, another significant SNP (S7_60258739; PVE = 4.1%; MAF = 0.12) was 60 kb from the sorghum ortholog (Sobic.007G168300) of the rice *SPINDLY* (*SPY*) gene. Across all vegetative traits QTL (for BLUPs and single environment mapping) (n = 114), 105 QTL (92%) did not colocalize with an a priori candidate gene, within the 250 kb LD window considered in this study.

### 3.4 Genome‐wide prediction accuracies

**FIGURE 4:**
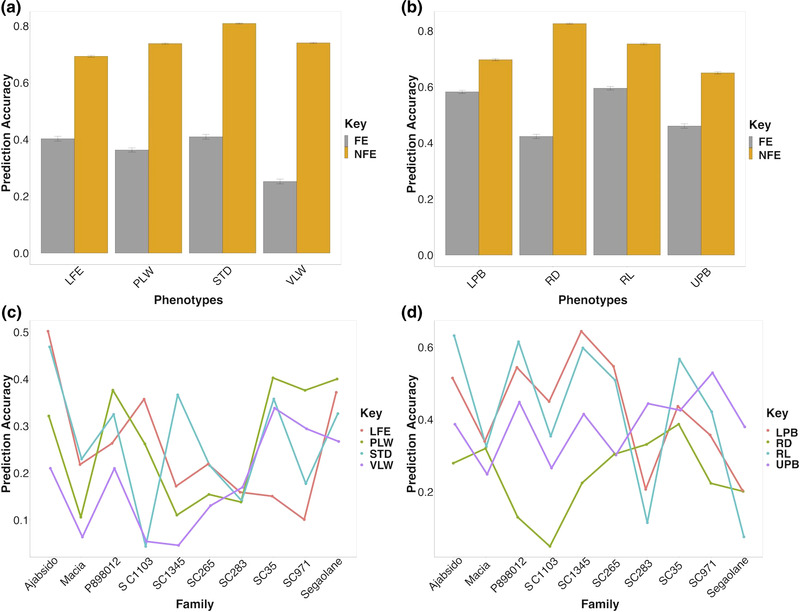
Genomic prediction accuracy for plant architecture traits. Upper panels are prediction accuracies from five‐fold cross‐validation for vegetative (a) and inflorescence (b) architecture traits. For “no family effect” (NFE) models, the response variables were the best linear unbiased predictors (BLUPs) of trait values. For the “family effect” (FE) models, the response variables were the residuals of trait value BLUPs, after a regression on family. Lower panels are prediction accuracies from leave‐one‐family‐out approach, plotted by family for vegetative (c) and inflorescence (d) architecture traits. For all panels the prediction accuracies are Pearson correlations between observed trait value BLUPs and predicted trait values. Trait abbreviations: leaf erectness (LFE), preflag leaf width (PLW), stem diameter (STD), vegetative leaf width (VLW), lower primary branch (LPB), rachis diameter (RD), rachis length (RL), upper primary branch (UPB)

To assess the potential for genome‐wide prediction of plant architecture, we cross‐validated genomic BLUPs of eight traits using two approaches (Figure 4). We contrasted genomic prediction accuracy for vegetative architecture traits with accuracies for inflorescence architecture traits. With the five‐fold cross‐validation approach, moderate to high prediction accuracies were observed for all the traits when the residuals obtained from regression of phenotypes on family (family‐effect model, FE; *r* = ∼0.2–0.6), When raw phenotypic data were used as the response variable in the genomic prediction model (no‐family‐effect model, NFE) accuracies were higher (*r* = ∼0.6–0.8). Mean prediction accuracy (across five folds and 100 cycles) for the FE model ranged from 0.25 in VLW to 0.41 in STD, and 0.42 in RD to 0.60 in RL. Mean prediction accuracy for the NFE model ranged from 0.69 in LFE to 0.81 in STD and 0.65 in UPB to 0.83 in RD (Figure 4a‐b). For the leave‐one‐family‐out approach, prediction accuracies were low to moderate, ranging from 0.10 to 0.6 across all traits (Figure 4c‐d). For the leave‐one‐family‐out approach, prediction accuracies were higher when certain families were used as validation sets (e.g., Ajabsido, P898012, SC35, and Segaolane). Inflorescence architecture traits were generally better predicted than vegetative architecture traits, other than RD, which was poorly predicted.

## 4 DISCUSSION

### 4.1 Genetic architecture of plant architecture in sorghum

The abundant variation in plant architecture in sorghum germplasm has long been noted (Vavilov, 1922), but the genetic architecture of this variation is not yet understood (Mullet, 2017). A main objective of this study was to characterize the genetic architecture of this variation using NAM. NAM revealed several loci that explain a moderate proportion of vegetative trait variation, which are segregating in global sorghum diversity. All QTL associated with leaf traits were of moderate to small effect, explaining less than 4% of phenotypic variation. In maize, small effect loci (explaining < 4% of phenotypic variation) were found for both leaf erectness and leaf width (Tian et al., 2011). Mating system (outcrossing versus selfing) of species shapes the genetic architecture of complex traits, with selfing species expected to harbor a greater proportion of large effect QTL than outcrossers (Buckler et al., 2009; Tian et al., 2011). Our findings suggest that the relationship between mating system and QTL effect size may be trait specific, since leaf morphology traits QTL in both sorghum and maize NAM explain < 4% of phenotypic variation.

A previous study of a biparental mapping population identified a colocalized LFE and plant height QTL on chromosome 7 (Hart, Schertz, Peng, & Syed, 2001) which may correspond to the QTL we observe around 60 Mb on chromosome 7 (Table 3). Earlier QTL mapping of leaf erectness in sorghum using biparental populations identified an average of four QTL per population, with most QTL having an effect size of about 15% (Truong, McCormick, Rooney, & Mullet, 2015). However, the estimated effect size of these loci may have been inflated due to the Beavis effect in these small mapping populations (Xu, 2003). The allelic effect estimates in this study are expected to be more accurate due to the large number of RILs in the NAM population (i.e., 2200 RILs) (King & Long, 2017). A positive relationship was observed between heritability and the number of associated QTL since only the two low heritable leaf width traits were the ones with the least QTL number (compare Table 2 and Table 3).

Pleiotropy is particularly important for ideotype breeding, since selection on one trait may inadvertently change the phenotypic mean of other traits that are under shared genetic controls (Price & Langen, 1992). The modest negative correlation (*r* = −0.10) between HGT and LFE suggests that short plants generally will have more erect leaves, which may facilitate adaptation for better light interception under high‐density planting. The positive relationship between FLT and STM, is consistent with the expectation that late‐flowering plants should have increased stem width due to the accumulation of biomass (Ashworth, Walsh, Flower, Vila‐Aiub, & Powles, 2016).

### 4.2 Candidate genes for plant architecture variation

NAM provides an approach to investigate potential genes underlying natural variation. While fine mapping is needed to positively identify causal genes, a lack of colocalization of a priori candidate genes with NAM QTL can provide evidence that the given genes do not underlie common variation. Given that several genes have been characterized to underlie natural variation in leaf and plant architecture in maize and rice, we hypothesized that sorghum orthologs of these genes also underlie leaf morphology and stem diameter variation in sorghum (Folta, 2015). However, we found no significant enrichment (*P*‐value = .19) of the QTL identified in this study with sorghum orthologs of a priori genes underlying variation in leaf morphology traits and stem diameter.

Despite a lack of significant enrichment for a priori candidate genes, a few of the significant associations in this study were located near candidate genes for both leaf morphology and plant architecture (Table 3; Supplemental Table S1). Some of these genes are known hormone transporters like the sorghum auxin transporter gene *Dw3* (Sobic.007G163800), which colocalized with both stem diameter and leaf erectness associated SNPs from single‐environment analysis. Tall *Dw3* revertants have significantly more stem biomass than their short *dw3* counterparts (George‐Jaeggli, Jordan, van Oosterom, Broad, & Hammer, 2013). Association studies in sorghum suggest a pleiotropic effect of *Dwarf3* on leaf erectness and inflorescence architecture (Brown, Rooney, Franks, & Kresovich, 2008). The colocalization of this gene alongside the negative correlation between stem diameter and plant height in this study suggests possible pleiotropic effect of the *Dw3* gene on stem diameter as previously observed for leaf erectness (Truong et al., 2015). In addition, two LFE QTL with the largest effect size among LFE QTL (PVE = 3.8–4.1%) colocalized (60 kb and 210 kb) with the putative sorghum ortholog (Sobic.007G168300) of the *SPY* gene, a negative regulator of gibberellin (Shimada et al., 2006).

The sorghum ortholog (Sobic.002G247800) of rice *Ideal Plant Architecture1* (*IPA1*, encoding an SPL transcription factor) colocalized with a common LFE‐associated SNP (S7_63865966, PVE = 1.5%, MAF = 0.19). In rice, increased transcript accumulation of *IPA1* (also known as *OsSPL14* and *WFP)* resulted in reduced tiller number, stronger culms, and denser panicles (Jiao et al., 2010). Pleiotropic effects of the sorghum ortholog of *IPA1* on stem diameter in sorghum is also plausible since a significant STD‐associated SNP colocalized with the sorghum ortholog of *IPA1* (200 kb away). Stem diameter is thought to be a component of lodging resistance and QTL for increased stem diameter have been used for the improvement of lodging resistance in rice (Kashiwagi et al., 2008). Another LFE QTL colocalized (132 kb away) with a putative sorghum ortholog (Sobic.001G445900) of the rice brassinosteroid biosynthesis gene *OsDWARF4*. *OsDWARF4* mutants have erect leaves that result in increased grain yields under high density planting (Sakamoto et al., 2006). Given the evidence that *liguleless* genes condition natural variation for leaf angle in maize (Tian et al., 2011) we considered the hypothesis that *liguleless* homologs also underlie leaf angle variation in sorghum. However, no LFE QTL colocalized with homologs of *lg* genes (Table 3), suggesting that in contrast to maize, *liguleless* genes do not underlie common natural variation for leaf angle in sorghum.

Near isogenic line development, fine mapping, and/or molecular cloning of these candidate genes would be required to test the hypotheses that they contribute to variation in sorghum. Overall, however, our observation that many sorghum QTL do not colocalize with homologs of canonical maize and rice vegetative development regulators (e.g., most QTL in Figure 3) suggests that much of the natural variation in vegetative morphology sorghum is due to genes not previously described in cereals. This finding is consistent with recent molecular cloning studies, which have revealed that while some classical sorghum genes are orthologs of canonical genes known from model crops (e.g., *Tannin2* [Wu et al., 2019], *Maturity6* [Murphy et al., 2014]), many others are novel genes (e.g., *Dwarf1* [Yamaguchi et al., 2016], *Maturity2* [Casto et al., 2019], *Dry* [Zhang et al., 2018]).

### 4.3 Prospects for genome‐wide prediction of plant architecture

The utility of genomic prediction for sorghum has been investigated in several recent studies (Hunt, van Eeuwijk, Mace, Hayes, & Jordan, 2018; Velazco et al., 2019). The high heritabilities and moderate prediction accuracies observed for each trait (Figure 4) suggest that genomic selection is possible for sorghum inflorescence morphology. This observation is despite the substantial oligogenic component which could make these traits less suited to genomic prediction models that assume polygenic variation (Bernardo, 2008). However, there is substantial variation in prediction accuracies obtained in the “leave‐one‐family‐out” approach. This family‐to‐family variation may be a reflection of the differences in genetic architecture among NAM families of different botanical types. Prediction accuracies also varied among families in the maize NAM population (Peiffer et al., 2014), consistent with among‐family differences in genetic architecture. The low prediction accuracies observed in some families (SC1103 for STD, RD, and VLW; SC1345 for VLW) suggests that these families may have variants or interactions which are not well‐represented in the remaining NAM families. A better understanding of epistatic background effects may be needed to understand among‐family variation in prediction accuracies (Blanc, Charcosset, Mangin, Gallais, & Moreau, 2006).

This genotype‐phenotype map for plant architecture in sorghum has several potential uses in crop improvement. Trait‐associated SNPs may be useful to develop molecular markers to facilitate simultaneous selection and improvement of multiple traits. However, attention has to be paid when selecting correlated traits that may have antagonistic effects on plant architecture or other aspects of agroclimatic adaptation (e.g., Figure 2). Finally, our study showed that the NAM population could be useful to train genomic predictions for breeding populations that share kinship with the NAM population founders.

## AUTHOR CONTRIBUTIONS

MOO and GPM conceived and designed the study. MOO and ZH collected the data. MOO analyzed the data. MOO and GPM wrote the manuscript. All authors edited and approved the manuscript.

## CONFLICT OF INTEREST

The authors declare no conflict of interest.

## Supporting information

SUPPORTING MATERIAL

SUPPORTING MATERIAL

## Data Availability

Genotype data are previously published and available at Dryad Digital Repository (https://doi.org/10.5061/dryad.gm073). Phenotype data are available in Supplemental File 1. Seed for the NAM population is available from the US National Plant Germplasm System.
