# Supplementary material for: Genome‐wide mapping and prediction of plant architecture in a sorghum nested association mapping population
Source: Plant Genome. 2020 Aug 7;13(3):e20038. doi: 10.1002/tpg2.20038 (PMC12806869; doi:10.1002/tpg2.20038)
Supplement: Supplementary file 1 [file TPG2-13-e20038-s002.docx]

## Supplementary Figures and Tables

**Figure S1: Phenotypic evaluation of leaf morphology.** Leaf erectness and leaf width measurements. A barcode protractor and barcode ruler were both used for the measurements of these traits to ensure accurate and fast data acquisition.

**Figure S2: Relationships among plant architecture traits by environment.** Pairwise Pearson correlation among traits in each environment. MN14.PFLW (preflag leaf width in environment 1), HI15.PFLW (preflag leaf width in environment 4), MN14.VLW (vegetative leaf width in environment 1), HI15.VLW (vegetative leaf width in environment 4), MN14.LFE (Leaf Erectness in environment 1), HI15.LFE (Leaf Erectness in environment 4), STD.MN14 (Stem Diameter in environment 1), Stem Diameter in environment 2 (STD.HI14). The correlation values shown are significant at *** *P*-value < 0.001, ** *P*-value < 0.01, and * *P*-value < 0.05.

**Table S1: Joint linkage mapping significant markers that colocalize with *a priori* candidate genes within 250 kb.**

| Trait | Marker | QTL^a^ | PVE^b^ | MAF^c^ | AES^d^ | Gene ID | Sorghum Ortholog | Distance (Kb) |
| --- | --- | --- | --- | --- | --- | --- | --- | --- |
| LFE.HI15 | S7_60108209 | qLFE7.601 | 3.8 | 0.12 | 6.3 | SPY | Sobic.007G168300 | 210 |
| LFE.MN14 | S1_72392397 | qLFE1.724 | 1.4 | 0.25 | -2.1 | DWARF4 | Sobic.001G445900 | 132 |
| LFE.MN14 | S7_60026279 | qLFE7.600 | 3.8 | 0.18 | 4.7 | DW3 | Sobic.007G163800 | 204 |
| LFE.MN14 | S7_63865966 | qLFE7.639 | 1.5 | 0.19 | 3.7 | IPA1 | Sobic.007G210200 | 120 |
| STD.HI14 | S10_8053746 | qSTD10.805 | 0.6 | 0.22 | 1.4 | BUL1 | Sobic.010G091700 | 46 |
| STD.MN14 | S7_59787744 | qSTD7.598 | 1.8 | 0.18 | 0.5 | DW3 | Sobic.007G163800 | 34 |
| STD.MN14 | S7_63675831 | qSTD7.637 | 1.2 | 0.3 | 0.1 | IPA1 | Sobic.007G210200 | 202 |
| STD.MN14 | S10_8142199 | qSTD10.814 | 1.2 | 0.22 | 1 | BUL1 | Sobic.010G091700 | 43 |

^e^ Stem Diameter in environment 1 (STD.MN14), STD.HI14 (Stem Diameter in environment 2), Leaf Erectness in environment 1, Leaf Erectness in environment 4.

^a^ QTL: Quantitative Trait Loci.

^b^ PVE: Proportion of variation explained.

^c^ MAF: Minor allele frequency.

^d^ AES: Additive Effect Size.

^d^ Gene ID: Abbreviated gene name. See text for details.
